# Reinforcement and Toughening of Thermo-Compressed Guar Gum Films with Untreated Rice Husk for Eco-Friendly Packaging Applications

**DOI:** 10.3390/polym18131558

**Published:** 2026-06-23

**Authors:** Theeraphol Phromsopha, Yodthong Baimark

**Affiliations:** Biodegradable Polymers Research Unit, Department of Chemistry and Centre of Excellence for Innovation in Chemistry, Faculty of Science, Mahasarakham University, Maha Sarakham 44150, Thailand; theeraphol.p@msu.ac.th

**Keywords:** guar gum, rice husk, biocomposite films, thermocompression molding, mechanical properties

## Abstract

This study investigates the fabrication of eco-friendly composite films based on guar gum (GG) reinforced with untreated rice husk (URH) powder (5–30 wt%) via a thermocompression process. To the best of our knowledge, this is one of the first demonstrations of directly utilizing untreated rice husk as a multifunctional reinforcing filler in GG-based bioplastics without any chemical or surface modification, thereby eliminating energy-intensive pretreatment steps. Particle dispersion and interfacial adhesion were optimal up to 10 wt% loading, above which agglomeration occurred. The incorporation of URH enhanced the thermal stability of the matrix. Mechanical performance peaked at 10 wt% URH, exhibiting a 90% increase in tensile strength, a 32% increase in elongation at break, and a 246% improvement in toughness compared to the neat GG film. Furthermore, URH addition reduced moisture content and water vapor permeability while increasing the water contact angle. Although film opacity increased, the results demonstrate that URH acts as an effective multifunctional filler. These GG/URH composite films exhibit strong potential for scalable industrial applications in eco-friendly food packaging, including disposable pouches and trays, offering a sustainable alternative to petroleum-based plastic materials.

## 1. Introduction

Plastic waste derived from fossil-based polymers has become a critical environmental issue due to the persistence of microplastics and nanoplastics in ecosystems [1,2,3]. To address this challenge, biodegradable polymers—particularly polysaccharides—have attracted increasing attention as sustainable alternatives for single-use packaging due to their abundance, renewability, and low environmental impact [4,5,6,7,8]. Among them, guar gum (GG), a galactomannan polysaccharide derived from *Cyamopsis tetragonoloba*, has been widely studied owing to its excellent film-forming ability, biodegradability, and biocompatibility [9]. GG has been applied in food packaging, drug delivery, wound dressings, biosensors, and wastewater treatment [10,11,12,13,14,15,16,17,18]. However, its practical application in packaging is limited by poor mechanical strength, high hydrophilicity, and limited water resistance [19,20,21,22], as well as a predominant reliance on solution casting methods, which are time-consuming and difficult to scale industrially [6,23].

To enhance the performance of guar gum (GG)-based films, a previous study has incorporated microcrystalline cellulose (MCC) as a reinforcing filler, resulting in improved mechanical strength and reduced moisture sensitivity [24]. However, MCC is derived through chemically intensive hydrolysis and purification processes involving strong acids and high energy input, leading to low production yield (30–40%) and limited scalability from both economic and environmental perspectives [25,26]. Therefore, although MCC is effective as a reinforcing phase, its sustainability is inherently constrained by multi-step chemical processing requirements.

In contrast, lignocellulosic agricultural residues such as rice husk (RH) have emerged as promising low-cost alternatives due to their abundance and minimal processing requirements. RH has been widely investigated as a reinforcing filler in both non-biodegradable polymers (e.g., polyethylene and polypropylene) and biodegradable matrices (e.g., thermoplastic starch and polylactic acid) [27,28,29,30,31,32]. Compared with MCC, RH offers additional multifunctionality arising from its intrinsic composition, including cellulose, hemicellulose, lignin, and a high silica content (~15–20 wt%), which collectively contribute to stiffness, thermal resistance, and barrier potential.

However, a critical limitation consistently reported in RH-based composites is the strong tendency toward particle aggregation at moderate-to-high loadings, driven by poor interfacial compatibility between the hydrophilic polymer matrices and the heterogeneous, wax- and lignin-covered surface of RH particles [33]. This results in stress concentration sites and deteriorated mechanical performance, particularly in systems relying on weak interfacial adhesion. Although surface modification strategies can partially mitigate these issues, they introduce additional chemical steps, increase processing cost, and undermine the environmental advantage of using agro-waste fillers [34].

Importantly, despite extensive studies on RH in conventional and biodegradable polymers, a direct comparison between chemically refined cellulose fillers (e.g., MCC) and untreated rice husk (URH) within guar gum matrices remains largely unexplored, particularly under thermomechanical processing routes. While prior studies have explored rice crop residues within multi-component, complex synthetic/natural blends (such as pectin/GG/polyvinylpyrrolidone matrices [35]), reports focusing specifically on the binary composite system of GG and URH powder prepared via melt-blending or industrial thermo-compression techniques remain extremely limited. Therefore, this study aims to fill this gap by fabricating binary GG/URH composite films using an industrially viable thermo-compression process. The chemical structure, thermal stability, crystalline patterns, phase morphology, tensile performance, water resistance, and optical properties of the fabricated films were systematically investigated to evaluate their viability as sustainable packaging materials.

## 2. Materials and Methods

### 2.1. Materials

**Figure 1:**
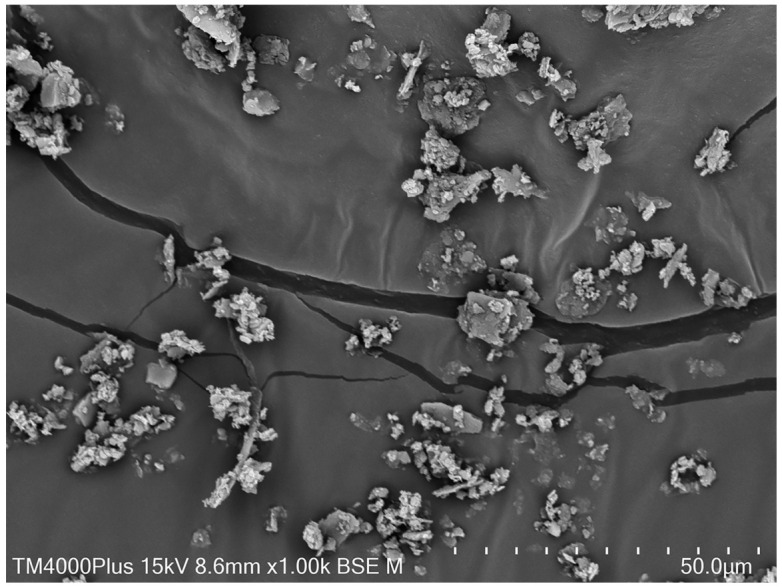
SEM micrograph of untreated rice husk (URH) powder. Scale bar = 50 µm.

Food-grade guar gum (GG) powder with a minimum galactomannan content of 82% and a viscosity of 6000 cps in a 1% aqueous solution was sourced from Chanjao Longevity Co., Ltd., in Bangkok, Thailand. Glycerol with a purity of 99.5% was obtained from QReC in Pathum Thani, Thailand. Rice husk (RH) was collected from a local rice mill located in Kalasin province, Thailand. Untreated rice husk (URH) was washed with distilled water, dried at 50 °C in an air-flow oven for 24 h, and ground using a Tefal food grinder (Moulinette Essential 300 W, Guangdong, China). The resulting powder was then sieved through a 200-mesh screen, ensuring that all particles used in this study were below 74 μm. The moisture content of URH was 2.8 ± 0.5 wt%. SEM analysis (Figure 1) confirmed the presence of irregularly shaped particles within this controlled microscale size range.

### 2.2. Preparation of GG/URH Composite Films

GG/URH composite films were prepared according to methodologies adapted from previous literature [24]. GG and URH (0, 5, 10, 20, and 30 wt% relative to GG) were mixed with an aqueous glycerol solution, manually kneaded, and granulated. The glycerol and total solution weights were maintained at 30 wt% and three times the GG weight, respectively (Table 1). Thermo-compression was performed on an Auto CH Carver molding machine (Wabash, IN, USA) at 120 °C for 5 min, followed by cooling for 5 min, maintaining a consistent pressure of 5 MPa throughout. These processing parameters were selected based on our previously established baseline protocols [24] to facilitate a direct, controlled comparison with prior filler studies. Specifically, prior screening indicated that 120 °C provides sufficient thermal softening to facilitate the flow of the plasticized GG matrix. The thickness of the fabricated films was measured using a digital micrometer at three random positions per specimen to ensure uniformity. The resulting films, exhibiting an average thickness within the range of 0.2–0.3 mm, were dried at 30 °C for 6 h in an air-flow oven and subsequently conditioned at 25 ± 2 °C and 50 ± 5% RH for 14 days before testing.

### 2.3. Characterization of GG/URH Composite Films

#### 2.3.1. FTIR Analysis

The functional groups of the sample films and URH powder were analyzed using the Invenio-S Bruker Fourier transform infrared (FTIR) spectrometer (Karlsruhe, Germany). This instrument is equipped with an attenuated total reflection (ATR) diamond attachment and operates within a wavenumber range of 500 to 4000 cm^−1^. The ATR-FTIR spectra were obtained by accumulating 32 scans and a resolution of 4 cm^−1^.

#### 2.3.2. Thermal Decomposition Properties

The thermal decomposition properties of the sample films and URH powder were determined using the SDT Q600 TA-Instruments thermogravimetric analyzer (TGA, New Castle, DE, USA) under a nitrogen atmosphere. The samples were heated from 50 to 800 °C at a rate of 20 °C·min^−1^.

#### 2.3.3. Crystal Structures

The crystal structures of the sample films and URH powder were investigated using the D8 Advance Bruker X-ray diffractometer (XRD, Karlsruhe, Germany) equipped with a Cu Kα radiation source operating at 40 kV and 40 mA. The XRD patterns were collected with a scan rate of 3° min^−1^. The relative degree of crystallinity (X_c_) of the fabricated films was calculated from the XRD profiles by decoupling the total crystalline and amorphous peak areas according to the following equation.

X_c_ (%) = [A_c_/(A_c_ + A_a_)] × 100 (1)

where A_c_ represents the integrated total area under the crystalline peaks, and A_a_ represents the integrated area of the amorphous halo.

#### 2.3.4. Phase Morphology

The phase morphology of the sample films and URH powder was analyzed using the TM4000Plus Hitachi scanning electron microscope (SEM, Tokyo, Japan) at an acceleration voltage of 15 kV. The film samples were cryofractured in liquid nitrogen. The samples were sputter-coated with gold before SEM analysis.

#### 2.3.5. Mechanical Properties

The mechanical performance of the composite films was evaluated using a universal testing machine (Model LY-1066B, Liyi, Dongguan, China) operated in tensile mode at 25 ± 2 °C and 50 ± 5% RH, equipped with a 100 kg load cell. Rectangular specimens (10 mm in width) were tested with a gauge length of 40 mm and a crosshead speed of 50 mm·min^−1^. The tensile toughness was determined by integrating the area under the stress–strain curves. Five replicates were tested for each formulation, and the results were reported as the average value ± standard deviation (SD).

#### 2.3.6. Surface Wettability

The surface wettability of the composite films was evaluated via the sessile drop method using an OCA11 contact angle analyzer (DataPhysics Instruments, Filderstadt, Germany) at 25 ± 2 °C and 50 ± 5% RH. A 2.5 µL droplet of deionized water was deposited onto the film surface, and the contact angles on both the left and right sides were measured after 15 s to ensure stabilization. Three measurements were performed at different locations for each sample, and the results were reported as the mean ± SD.

#### 2.3.7. Moisture Content

The moisture content of the composite films was determined by the weight loss method. Film specimens (20 mm × 20 mm) were weighed to obtain the initial weight (*W*_1_) and then dried in an oven at 105 °C for 24 h to reach a constant weight. The dried samples were reweighed to determine the final weight (*W*_2_). The moisture content was calculated using the following equation:

Moisture content (%) = [(*W*_1_ − *W*_2_)/*W*_1_] × 100 (2)

Three replicates were tested for each formulation, and the results are presented as the mean ± SD.

#### 2.3.8. Water Vapor Permeability

The water vapor permeability of the composite films was determined using the desiccant method [36]. The film sample was used to seal a glass bottle containing 10 g of anhydrous calcium chloride (0% RH). The effective permeation area was 254.34 mm^2^. The sealed glass bottles were placed in a desiccator maintained at 25 °C and 75% relative humidity (RH) using a saturated sodium chloride solution. The weight gain of the glass bottles was recorded every 24 h over a period of 7 days. The water vapor transmission rate (WVTR) was determined from the slope of the weight change vs. time curve using linear regression (*r*^2^ > 0.99). The WVP (g·mm^−1^·h^−1^·Pa^−1^) was then calculated using the following formula:

WVP (g·mm^−1^·h^−1^·Pa^−1^) = (Slope × d)/(A × ΔP) (3)

where d is the film sample thickness (mm), A is the film area (mm^2^), and ΔP is the film’s vapor pressure differential (1753.55 Pa) [37]. Measurements were performed in triplicate, and the results were reported as mean ± SD.

#### 2.3.9. Film Thickness and Opacity

The thickness of the composite films was measured using a digital micrometer (Mitutoyo, Kanagawa, Japan) with an accuracy of 0.001 mm. The opacity of the films was determined by measuring the absorbance at 600 nm (A_600_) using a UV-Vis spectrophotometer (Cary 60, Agilent Technologies, Victoria, Australia). The film opacity (mm^−1^) was calculated according to the following equation [38]:

Film opacity (mm^−1^) = A_600_/*X* (4)

where A_600_ is the absorbance at 600 nm and *X* is the average film thickness (mm). For each formulation, measurements were performed in triplicate, and the results were reported as mean ± SD.

### 2.4. Statistical Analysis

Statistical significance was evaluated using one-way analysis of variance (ANOVA), followed by Duncan’s multiple range test for post hoc comparison of means. All analyses were performed using SPSS software (version 22.0). A *p*-value of less than 0.05 was considered statistically significant. Data are presented as the mean ± SD.

## 3. Results and Discussion

### 3.1. FTIR Analysis

**Figure 2:**
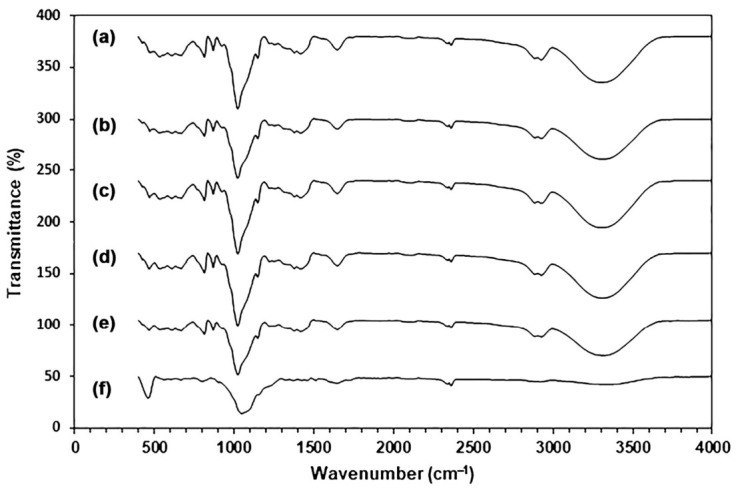
FTIR spectra of (**a**) neat GG film; GG/URH composite films with URH contents of (**b**) 5 wt%, (**c**) 10 wt%, (**d**) 20 wt%, and (**e**) 30 wt%; and (**f**) URH powder.

The chemical structures of the film samples and pristine URH powder were evaluated via FTIR spectroscopy, as illustrated in Figure 2. The specific peak assignments and their corresponding vibrational modes are systematically organized in Table 2. The neat GG films [Figure 2a] exhibited characteristic peaks corresponding to O–H stretching vibrations [39], asymmetric C–H stretching of methylene groups within the polymer backbone [39], and C–O–C stretching of the glycosidic linkages in the galactomannan structure [40,41]. The broadness of the O–H band signifies extensive hydrogen bonding networks formed among the hydroxyl groups of the GG matrix, glycerol molecules, and bound water [42]. For the URH powder [Figure 2f], the spectrum displayed typical lignocellulosic features, including O–H stretching, aliphatic C–H stretching, and C=O stretching of carbonyl groups [43,44]. Notably, the presence of amorphous silica within the URH structure was confirmed by the distinct siloxane (Si–O–Si) stretching bands, as well as symmetric Si–O–Si stretching and Si–O bending vibrations in the lower wavenumber region [45,46].

Prior research on GG/MCC composite films demonstrated strong intermolecular hydrogen bonding evidenced by a shift in O–H stretching bands [24]. In contrast, GG/URH composites showed no significant FTIR peak shifts compared to neat GG, indicating the absence of newly formed strong chemical interactions. However, this does not exclude weak interfacial interactions that may arise from physical contact between phases, particularly in heterogeneous particulate systems. This behavior can be reasonably attributed to two main factors. First, glycerol, as a highly effective plasticizer, strongly interacts with GG chains via hydrogen bonding, thereby dominating the available hydroxyl interaction sites within the matrix and limiting additional specific interactions with URH particles. Second, the surface of untreated rice husk is partially covered by lignin, waxy components, and silica-rich layers [47,48], which reduce the accessibility of internal cellulose hydroxyl groups and hinder direct molecular-level interactions with the GG matrix.

Therefore, the reinforcement mechanism in GG/URH films is more likely governed by physical interactions, including physical entrapment and micro-mechanical interlocking, as further supported by SEM observations and thermal behavior analysis in this study. This interpretation is consistent with previous reports on untreated lignocellulosic-filled polymer systems, where FTIR spectra typically show minimal changes despite the presence of interfacial adhesion [49,50].

### 3.2. Thermal Decomposition Properties

**Figure 3:**
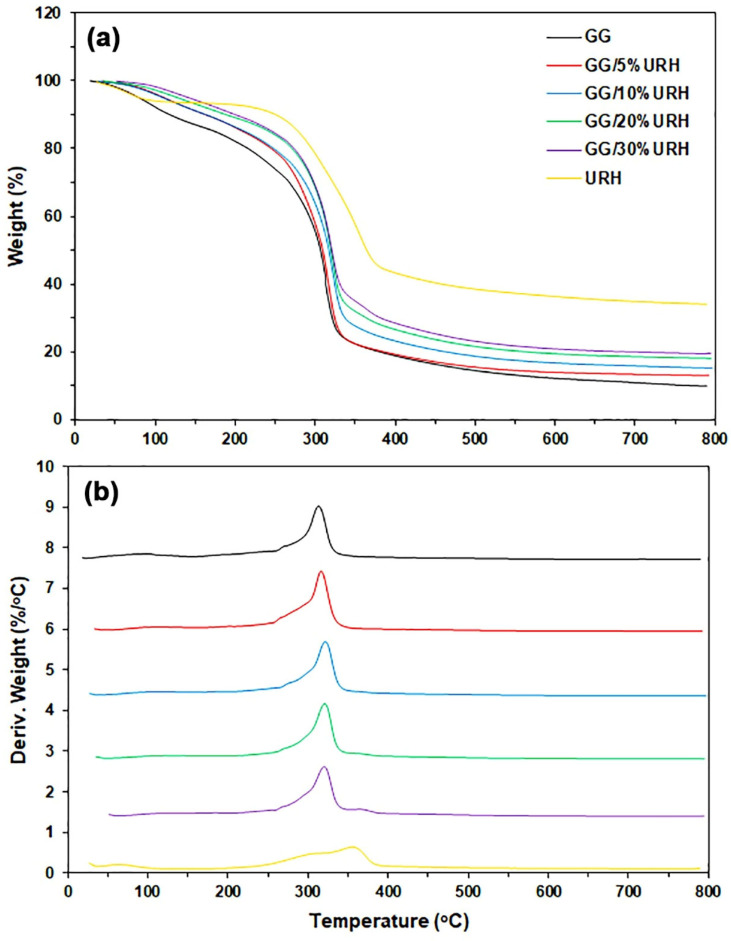
(**a**) TG and (**b**) DTG thermograms of neat GG film, GG/UHR composite films with various URH contents, and URH powder.

The thermal stability of film samples and URH was investigated using the thermogravimetric analysis (TGA). The thermogravimetric (TG) and derivative TG (DTG) thermograms of the samples are shown in Figure 3a and Figure 3b, respectively. The TGA results are summarized in Table 3. The TG thermogram of the URH-free GG films displayed two weight-loss stages within the temperature ranges of 50–150 °C and 150–400 °C, which are attributed to the evaporation of residue moisture and the decomposition of GG, respectively [51,52]. Temperatures corresponding to 5% weight loss (T_5%_) and 10% weight loss (T_10%_) of the URH-free GG films, due to moisture evaporation, are 79 °C and 118 °C, respectively. The temperature at 50% weight loss (T_50%_) is 307 °C, which is associated with the decomposition of the GG matrix. The URH showed two weight-loss stages in the temperature ranges of 50–150 °C and 200–400 °C, attributed to the evaporation of volatile substances and decomposition of hemicellulose and cellulose, respectively [53].

For the GG/URH composite films, the T_5%_ and T_10%_ values shifted to higher temperatures with increasing URH content compared to neat GG films, indicating a reduced initial weight loss associated with moisture and low-molecular-weight volatiles. The similar T_5%_ values of URH (80 °C) and neat GG (79 °C) suggest that early-stage mass loss is primarily governed by residual moisture rather than filler decomposition. The improvement in thermal stability of the composite films is attributed to the synergistic effects of URH constituents. In particular, the high amorphous silica content (~15–20 wt%) acts as an inorganic thermally stable phase, forming a physical barrier that restricts the diffusion and release of volatile degradation products [54]. In addition, lignin contributes to char formation over a broad temperature range, resulting in a thermally stable carbonaceous residue [35]. The combined silica-rich residue and lignin-derived char layer effectively retards heat and mass transport, thereby slowing the thermal decomposition of the GG matrix [55,56].

As a result, the T_50%_ values of GG/URH composites increased with URH loading, reflecting improved thermal resistance of the matrix. This interpretation is further supported by the increase in char residue at 800 °C (from 9.9 wt% for neat GG to significantly higher values for composites). The temperature at maximum decomposition (T_max_) also shifted from 313 °C for neat GG to 316–322 °C for GG/URH composites, confirming enhanced thermal stability. Compared with previous GG/MCC composites, which showed only a marginal increase of 1–4 °C in T_max_ [24], the present GG/URH system exhibited a more pronounced improvement (3–9 °C), indicating a stronger thermal stability effect of URH. This enhanced performance is mainly attributed to the higher silica and lignin content of URH, which provides superior thermal resistance compared to MCC.

### 3.3. Crystalline Structures

**Figure 4:**
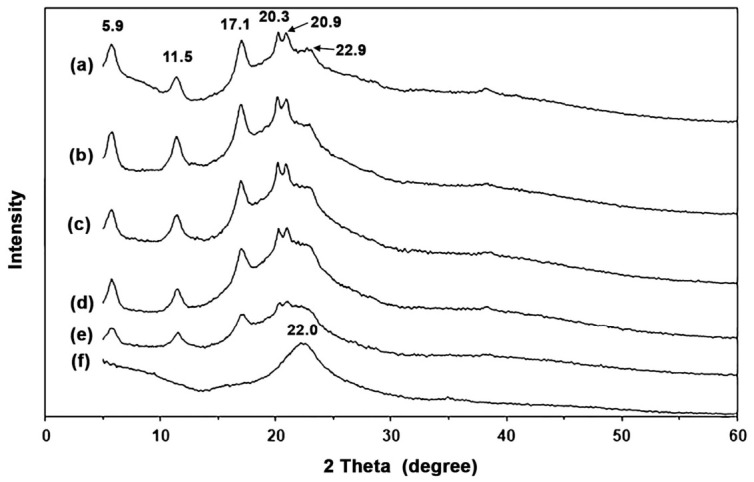
XRD patterns of (**a**) neat GG film; GG/URH composite films with URH contents of (**b**) 5 wt%, (**c**) 10 wt%, (**d**) 20 wt%, and (**e**) 30 wt%; and (**f**) URH powder.

As presented in Figure 4, X-ray diffraction (XRD) patterns were employed to examine the crystalline structures of the sample films. The glycerol-plasticized GG film exhibited characteristic diffraction peaks at 2θ values of 5.9°, 11.5°, 17.1°, 20.3°, 20.9°, and 22.9° [Figure 4a]. These diffraction features are associated with the enhanced chain rearrangement and crystallization of GG during the thermo-compression process facilitated by glycerol plasticization [24]. The URH sample exhibited a broad diffraction peak centered at 2θ = 22.0° [Figure 4e], corresponding to the (002) reflection of cellulose I crystallites [57]. However, this diffraction feature partially overlapped with the broad amorphous silica halo originating from the silica component of URH, which appeared in the range of 2θ = 18.0–22.0° [58].

The XRD patterns of the GG/URH composite films exhibited diffraction profiles that were similar to those of the neat GG film. All major diffraction peaks were aligned comparably, with no observable peak shifts. This indicates that the incorporation of URH did not result in detectable changes to the fundamental crystalline phase or polymorphic structure of the GG matrix. Furthermore, the characteristic diffraction peak of URH at approximately 2θ = 22.0° became more pronounced with increasing URH content, thereby confirming the successful integration of URH into the composite films.

To provide quantitative insight, the relative degree of crystallinity (X_c_) was calculated using Equation (1). The calculated X_c_ values were 16.16%, 18.84%, 18.93%, 11.94%, and 12.29% for the neat GG, GG/5%URH, GG/10%URH, GG/20%URH, and GG/30%URH films, respectively. At lower filler loadings (5 and 10 wt%), the crystallinity remained comparable to or exhibited a slight increase compared with that of the neat GG film, suggesting that well-dispersed URH particles may have provided favorable nucleation sites and promoted local GG chain ordering. However, increasing the URH content beyond 10 wt% resulted in a pronounced reduction in crystallinity. This decrease was likely associated with the aggregation of URH particles at higher loading levels, as observed from SEM analysis. Similar effects have been reported in polymer nanocomposite systems, where filler dispersion and interfacial interactions can influence polymer chain mobility and crystallization behavior [59]. Accordingly, the aggregation of URH particles may have imposed physical constraints on GG chains, restricting their rearrangement and packing. These physical constraints may have hindered the formation of ordered crystalline regions during the film-forming process.

### 3.4. Phase Morphology

**Figure 5:**
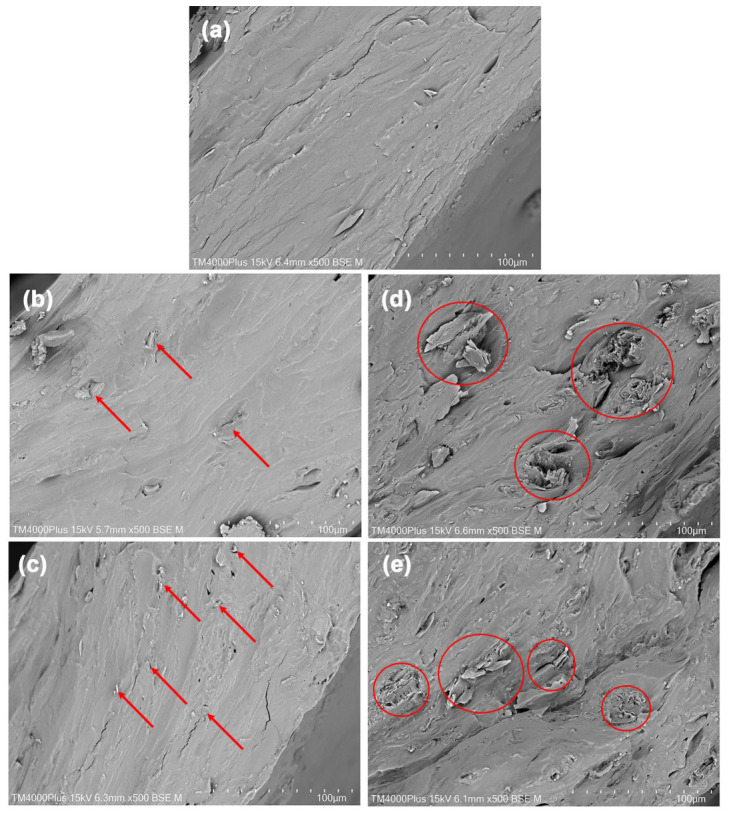
SEM micrographs of cryofractured surfaces of (**a**) neat GG film and GG/URH composite films with URH contents of (**b**) 5 wt%, (**c**) 10 wt%, (**d**) 20 wt%, and (**e**) 30 wt%. Red arrows indicate well-dispersed URH particles, while red circles highlight URH aggregates at higher loadings. All scale bars = 100 µm.

We examined the phase morphology of the film samples by capturing SEM images of their cryofractured surfaces, as shown in Figure 5. The GG films without URH exhibited smooth fracture surfaces with very few visible GG particles. This observation indicates that the glycerol solution effectively plasticized the GG, facilitating the formation of a smooth and continuous film through the coalescence of softened GG particles during thermo-compression molding [60]. In contrast, the GG/URH composite films revealed URH particles embedded within the GG matrix. Although minor micro-voids were observed at certain interfaces, no large or extensive gaps were present, indicating satisfactory interfacial adhesion between the URH particles and the GG phase. This intimate interfacial contact is primarily attributed to the thermo-compression molding process. Under thermal and mechanical pressure, the heated and softened glycerol-plasticized GG matrix can flow into the micro-crevices and surface irregularities of the URH particles. Upon cooling, the solidified GG matrix encapsulates the filler, generating a physically interlocked interface. This physical entrapment contributes to adequate interfacial bonding despite the lack of clear evidence for newly formed intermolecular hydrogen bonds between the two phases, as indicated by FTIR analysis. The integrity of this interface is further supported by the tensile performance discussed in the subsequent section, where efficient stress transfer across the filler–matrix boundary contributed to improved mechanical integrity and delayed interfacial failure during deformation.

The SEM micrographs reveal that the URH particles were relatively well dispersed at loadings of 5 wt% and 10 wt%, as indicated by the red arrows [Figure 5b and Figure 5c, respectively]. Conversely, noticeable particle agglomeration occurred at higher loadings of 20 wt% and 30 wt% [Figure 5d and Figure 5e, respectively, as highlighted by the red circles]. This aggregation behavior is likely driven by the inherent hydrophilic–hydrophobic incompatibility between the hydrophilic GG matrix and URH particles containing hydrophobic components, such as lignin and natural waxy coatings [49]. This observation is consistent with previous studies reporting filler agglomeration at increased loading levels [29]. The improved interfacial contact at optimal URH loadings may contribute to more effective stress and heat transfer between the GG matrix and rigid URH particles, thereby enhancing the structural integrity and thermal stability of the composite films, as reflected in the TGA results. Furthermore, these morphological characteristics provide a structural explanation for the variation in tensile properties, which are discussed in the subsequent section.

### 3.5. Mechanical Properties

**Figure 6:**
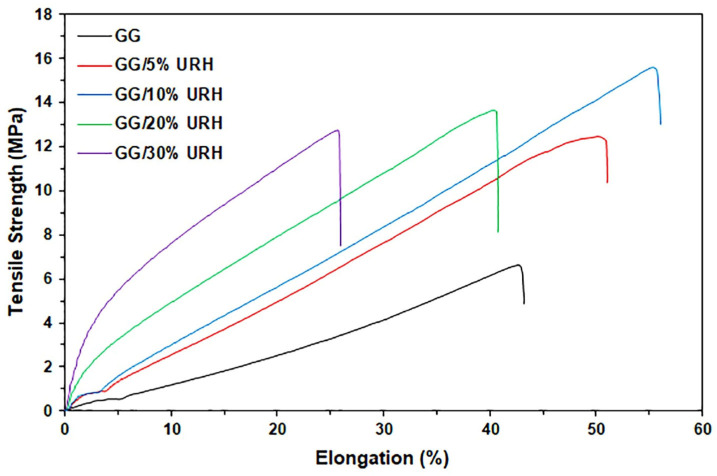
Selected tensile curves of GG and GG/URH composite films.

The mechanical properties of the film samples were evaluated via tensile testing, with the resulting stress–strain curves, Young’s modulus, tensile strength, elongation at break, and toughness summarized in Figure 6 and Table 4. The GG films without URH exhibited a tensile strength of 8.2 MPa, an elongation at break of 42.4%, and a Young’s modulus of 8.6 MPa. Upon incorporation of URH, the mechanical performance of the composite films improved significantly, reaching the highest tensile strength at a URH content of 10 wt% (15.6 MPa).

The Young’s modulus of the GG/URH composite films exhibited a substantial increase with increasing URH content, rising from 8.6 MPa for the neat GG film to 122.9 MPa for the GG/30 wt% URH film. This enhancement in stiffness is mainly attributed to the rigid filler effect of URH, which contains inorganic silica components together with lignocellulosic constituents. The presence of rigid URH particles within the plasticized GG matrix can restrict polymer chain mobility during tensile deformation, thereby increasing resistance to elastic deformation and improving the stiffness of the composite films.

Interestingly, despite the significant improvement in mechanical properties, the FTIR spectra discussed previously showed no clear shifts in the characteristic absorption bands. This suggests that the reinforcement mechanism was predominantly associated with physical encapsulation, mechanical interlocking, and structural anchoring of URH particles within the plasticized GG matrix rather than detectable chemical interactions [61,62]. During thermo-compression molding, the softened glycerol-plasticized GG matrix can flow into the surface irregularities and micro-crevices of untreated URH particles. Upon cooling, the solidified GG matrix physically anchors the filler particles, enabling effective stress transfer across the filler–matrix interface without substantially altering the molecular vibration environment, which is consistent with observations reported for untreated natural fiber-reinforced composites [63].

Comparative analysis indicates that the tensile strength and Young’s modulus of the GG/URH films were higher than those previously reported for GG/MCC composites at comparable filler loadings (5–30 wt%) [24]. For example, at 10 wt% filler loading, the GG/URH films achieved a tensile strength of 15.6 MPa and a Young’s modulus of 51.4 MPa, whereas the corresponding GG/MCC composite exhibited a tensile strength of 10 MPa and a Young’s modulus of 26 MPa. This improved reinforcing efficiency may be attributed to the combined contribution of the inorganic silica component and the rigid lignocellulosic structure of URH [58], which provide additional structural reinforcement compared with MCC-based fillers.

However, when the URH content exceeded 10 wt%, the tensile strength, elongation at break, and toughness (calculated from the area under the tensile curves) gradually decreased. At 30 wt% URH, the tensile strength decreased to 12.6 MPa, while the toughness decreased to 2.15 × 10^6^ J/m^3^. As supported by SEM observations, this reduction was likely associated with increased URH particle aggregation at higher filler loadings. The inherent hydrophilic–hydrophobic incompatibility between the hydrophilic GG matrix and the hydrophobic components of URH, including lignin and natural waxy coatings, may promote particle clustering [33,64]. These aggregated regions can act as local stress concentration sites, facilitating micro-crack initiation and accelerating premature fracture during tensile deformation. Nevertheless, even at high URH loadings (20–30 wt%), the composite films maintained higher tensile strength and toughness than the neat GG film (8.2 MPa and 1.28 × 10^6^ J/m^3^, respectively), demonstrating the reinforcing capability of URH within the GG matrix.

Regarding ductility, the elongation at break increased from 42.4% for the neat GG film to a maximum of 56.1% at 10 wt% URH before decreasing at higher filler loadings. This temporary improvement in deformability at low URH concentrations (≤10 wt%) may be related to improved filler dispersion and localized changes in the GG matrix structure. Well-dispersed URH particles may disturb local chain packing and reduce physical constraints within the GG network, allowing greater chain rearrangement during tensile deformation. In addition, rigid URH particles may also contribute to crack deflection and delay the growth of microcracks, which could improve the balance between strength and ductility. Overall, the system may be considered a hard-particle-in-soft-matrix architecture, which can enhance mechanical performance without requiring strong chemical bonding between the two phases [65,66].

It can be noted that certain formulations exhibited relatively large standard deviations in their tensile properties. This behavior is commonly observed in biomass-filled biocomposites and can be attributed to the intrinsic heterogeneity of rice husk particles, variations in filler dispersion state, and the possible formation of localized micro-voids within the matrix during thermo-compression molding. In addition, minor structural non-uniformities among individual specimens may contribute to differences in stress distribution and crack initiation behavior. Collectively, these factors lead to variations in load transfer efficiency at the filler–matrix interface, resulting in the observed scattering in tensile strength and ductility.

To evaluate the potential application of the developed films, their mechanical properties were compared with those of conventional packaging materials. Commercial low-density polyethylene (LDPE) films typically exhibit tensile strengths in the range of 10–13 MPa [67,68]. The maximum tensile strength achieved by the GG/URH composite films (15.6 MPa) exceeds this range, indicating that these biodegradable composite films possess mechanical properties comparable to or better than typical LDPE packaging films. These results demonstrate the potential of GG/URH composites as sustainable alternatives for packaging applications where adequate mechanical strength is required.

### 3.6. Surface Wettability, Moisture Content, and Water Vapor Permeability

**Figure 7:**
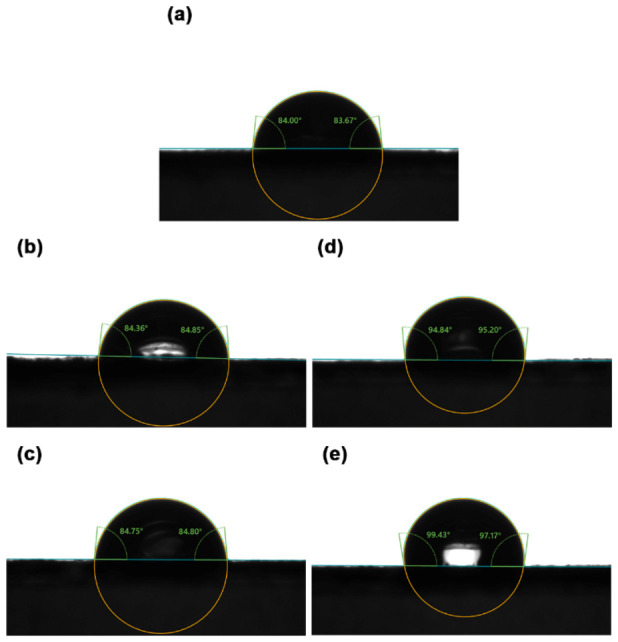
Images of water droplets and water contact angles on the surfaces of (**a**) neat GG film and GG/URH composite films with URH contents of (**b**) 5 wt%, (**c**) 10 wt%, (**d**) 20 wt%, and (**e**) 30 wt%.

The water resistance properties of the composite films were evaluated by examining their surface wettability, moisture content, and water vapor permeability. The water contact angles (WCAs) of the film samples were measured to characterize their surface wetting behavior. Figure 7 presents the WCA profiles of the different formulations, and the corresponding quantitative values are summarized in Table 5. A higher WCA value indicates reduced surface wettability and enhanced hydrophobic characteristics. The neat GG film exhibited an average WCA of 84.24°, reflecting the relatively hydrophilic nature of the glycerol-plasticized GG matrix. After incorporation of URH, the average WCAs of the films containing 5 wt% (85.12%) and 10 wt% URH (85.53%) remained statistically comparable to that of the neat GG film. However, further increasing the URH content beyond 10 wt% resulted in a significant increase in WCA, indicating improved surface hydrophobicity of the composite films. This enhancement is likely attributed to the introduction of hydrophobic components naturally present in untreated rice husk, particularly waxy substances and lignin, which can reduce surface affinity toward water. In addition, the presence of rigid silica-containing structures and increased surface heterogeneity from URH particles may contribute to reduced water droplet spreading, thereby further enhancing the apparent hydrophobicity of the composite surfaces [50].

As detailed in Table 5, the incorporation of URH gradually reduced the moisture content of the GG/URH composite films. This reduction may be attributed to the partial replacement of the highly hydrophilic GG matrix with URH components, including lignin, waxy substances, and inorganic silica, which exhibit different interactions with water compared with the GG phase. However, compared with previously reported GG/MCC films, the GG/URH composites exhibited higher moisture retention at comparable filler loadings. This difference is primarily attributed to the compositional and structural differences between URH and MCC. URH retains amorphous hemicellulose and other lignocellulosic components, which provide more accessible hydrophilic sites and may contribute to greater moisture retention compared with the highly purified crystalline cellulose structure of MCC [69]. In addition, ground rice husk particles exhibit an irregular and porous morphology with residual cellular structures and lumen-like cavities, unlike the relatively compact fibrous morphology of MCC, allowing greater physical retention of water molecules within the composite films [70].

Water vapor permeability (WVP) is an important parameter for food packaging applications, as reduced moisture transmission is essential for preserving food quality and extending shelf life. As summarized in Table 5, the neat GG film exhibited an average WVP value of 3.09 × 10^−9^ g·mm^−1^·h^−1^·Pa^−1^. After incorporation of URH, the WVP values of the films containing 5 wt% (2.91 × 10^−9^ g·mm^−1^·h^−1^·Pa^−1^) and 10 wt% URH (3.00 × 10^−9^ g·mm^−1^·h^−1^·Pa^−1^) remained statistically comparable to that of the neat GG film. However, further increasing the URH loading beyond 10 wt% resulted in a significant reduction in WVP, indicating enhanced resistance to water vapor transmission.

The improved barrier performance is likely associated with the structural characteristics of URH and its interaction within the GG matrix. As demonstrated by XRD analysis, URH contains cellulose type I crystalline domains along with silica-containing regions, which can contribute to the formation of rigid and less permeable domains within the composite structure. These rigid components, together with the lignocellulosic framework of URH, act as physical barriers that increase the complexity of the diffusion pathway. Consequently, water vapor molecules are required to travel through a more tortuous route within the composite matrix, reducing their effective transport rate [6]. Furthermore, the irregular morphology and heterogeneous structure of URH particles observed from SEM analysis may additionally contribute to the formation of a more complex diffusion pathway.

The enhanced WVP resistance is also consistent with the increased surface hydrophobicity observed from WCA measurements at higher URH loadings. The presence of naturally occurring hydrophobic waxy substances and lignin in untreated rice husk may reduce the surface affinity toward water, thereby limiting moisture interaction with the composite films. Therefore, the combined effects of the crystalline/inorganic domains identified by XRD, the tortuous diffusion pathway generated by URH particles, and the reduced surface water affinity contribute to the improved water vapor barrier properties of the GG/URH composite films.

### 3.7. Film Thickness and Opacity

The film thickness and opacity values of the prepared formulations are summarized in Table 6. Regarding thickness uniformity, all formulations showed acceptable thickness consistency, with standard deviations ranging from 0.03 to 0.07 mm. The relatively smaller deviations observed at higher URH loadings may indicate improved thickness uniformity, which could be associated with the increased solid content and reduced deformation of the composite matrix during thermo-compression molding. Furthermore, the presence of dispersed URH particles increases the resistance to deformation of the softened GG matrix, resulting in less thickness reduction under the applied compression conditions. Similar behavior has been reported in filler-reinforced polymer composite systems, where rigid particles restrict matrix deformation through filler–matrix interactions [27,71].

The film thickness and opacity values of the prepared formulations are summarized in Table 6. The incorporation of URH influenced the optical properties of the GG/URH composite films, particularly at higher filler loadings. The opacity values of the films containing 5 wt% (3.03 ± 0.12 mm^−1^) and 10 wt% URH (3.29 ± 0.21 mm^−1^) remained statistically comparable to that of the neat GG film (3.15 ± 0.10 mm^−1^). However, further increasing the URH content to 20 and 30 wt% significantly increased the opacity values to 4.22 ± 0.28 and 4.83 ± 0.14 mm^−1^, respectively. These results indicate that a higher concentration of URH particles substantially reduced light transmission through the composite films.

**Figure 8:**
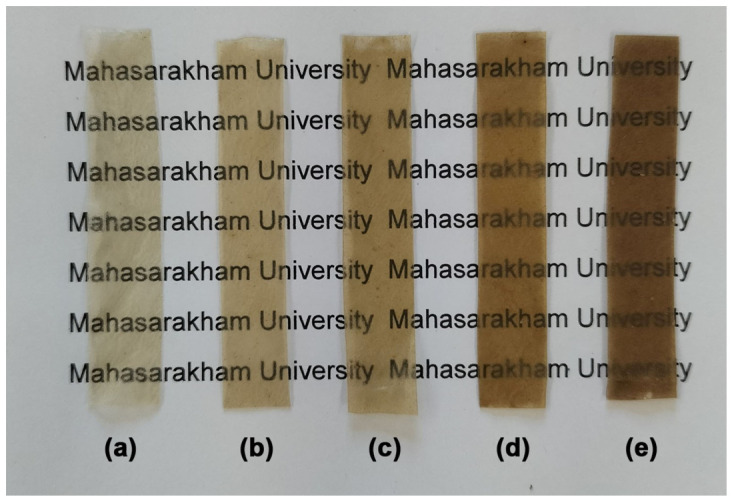
Visual appearance and transparency of (**a**) neat GG film and GG/URH composite films with URH contents of (**b**) 5 wt%, (**c**) 10 wt%, (**d**) 20 wt%, and (**e**) 30 wt%.

The increase in opacity at elevated URH loadings can be attributed to enhanced light scattering and blocking effects caused by the increased number of dispersed lignocellulosic filler particles within the GG matrix. The heterogeneous structure of URH, consisting of cellulose-rich domains, lignin, and silica-containing components, introduces additional internal interfaces that interfere with light propagation through the film. This interpretation is consistent with the visual observations shown in Figure 8, where the underlying text became increasingly obscured as the URH content increased. Nevertheless, the text remained visible for films containing up to 20 wt% URH, suggesting that the composite films maintained sufficient visual transparency for potential packaging applications requiring product visibility.

## 4. Conclusions

Eco-friendly GG/URH composite films were successfully fabricated via a thermocompression process using URH as a sustainable reinforcing filler. The reinforcement mechanism was dominated by physical entrapment and mechanical interlocking within the plasticized GG matrix. The mechanical and barrier properties were strongly dependent on URH loading. An optimum performance was achieved at 10 wt% URH, providing the highest tensile strength and toughness, while higher loadings (>10 wt%) induced particle agglomeration and reduced mechanical performance. In contrast, water vapor barrier properties continuously improved with increasing URH content due to the tortuous diffusion pathway created by silica-rich and lignocellulosic phases. This study is limited to laboratory-scale processing, and long-term durability and scale-up validation remain unaddressed. Future work will focus on biodegradation behavior and gas barrier performance (O_2_ and CO_2_) for real packaging applications. The use of URH without chemical modification highlights a cost-effective and scalable strategy for sustainable food packaging materials.

## Figures and Tables

**Table 1:** Formulations and sample designations of neat GG and GG/URH composite films with varying URH contents.

Sample Code	URH Content (wt%)	GG (g)	URH (g)	Glycerol (g)	Distilled Water (g)
GG	-	10.00	-	4.29	25.71
GG/5%URH	5	9.50	0.50	4.07	24.43
GG/10%URH	10	9.00	1.00	3.86	23.14
GG/20%URH	20	8.00	2.00	3.43	20.57
GG/30%URH	30	7.00	3.00	3.00	18.00

**Table 2:** FTIR peak assignments for neat GG film, URH powder, and GG/URH composite films.

Functional Group/ Vibration Type	GG (cm^−1^)	URH (cm^−1^)	GG/URH Composites (cm^−1^)	Relevant References
O–H stretching (hydroxyl groups, bound water)	3314	3355	3314	[39,43,44]
Asymmetric C–H stretching (methylene/aliphatic groups)	2922	2928	2922	[39,43,44]
C=O stretching (carboxylate/carbonyl groups)	–	1648	–	[44]
C–O–C/Si–O–Si stretching (glycosidic linkages/siloxane overlapping)	1020	1049	1020	[45,46]
Symmetric Si–O–Si stretching (amorphous silica)	–	802	–	[45,46]
Si–O bending (amorphous silica)	–	460	–	[45,46]

**Table 3:** Thermal degradation parameters of neat GG film, URH powder, and GG/URH composite films obtained from TGA and DTG analysis.

Sample	T_5%_ (°C) ^1^	T_10%_ (°C) ^1^	T_50%_ (°C) ^1^	Char Residue at 800 °C (%) ^1^	GG-T_max_ (°C) ^2^	URH-T_max_ (°C) ^2^
GG	79	118	307	9.9	313	-
GG/5%URH	110	162	309	13.1	316	-
GG/10%URH	111	163	317	15.3	322	-
GG/20%URH	128	189	319	18.2	321	-
GG/30%URH	142	200	321	19.6	321	-
URH	80	251	364	34.2	-	355

^1^ Obtained from TG thermograms. ^2^ Obtained from DTG thermograms.

**Table 4:** Mechanical properties of neat GG and GG/URH composite films with different URH contents.

Sample	Maximum Tensile Strength (MPa)	Elongation at Break (%)	Young′s Modulus (MPa)	Tensile Toughness (×10^6^ J/m^3^)
GG	8.2 ± 1.4 ^a^	42.4 ± 5.1 ^b^	8.6 ± 2.6 ^a^	1.28 ± 0.14 ^a^
GG/5%URH	12.5 ± 2.1 ^b^	51.1 ± 4.7 ^c^	22.3 ± 5.1 ^b^	3.27 ± 0.26 ^c^
GG/10%URH	15.6 ± 1.8 ^c^	56.1 ± 5.8 ^d^	26.1 ± 4.8 ^b^	4.43 ± 0.24 ^d^
GG/20%URH	13.8 ± 2.2 ^b^	41.2 ± 4.3 ^b^	65.1 ± 7.5 ^c^	3.22 ± 0.18 ^c^
GG/30%URH	12.6 ± 1.6 ^b^	26.7 ± 3.9 ^a^	112.9 ± 9.2 ^d^	2.15 ± 0.31 ^b^

Data are expressed as mean ± standard deviation (*n* = 5). Different superscripts (a, b, c, d) within the same column indicate significant differences (*p* < 0.05).

**Table 5:** Surface hydrophobicity, moisture content, and water vapor barrier properties of neat GG and GG/URH composite films.

Sample	Water Contact Angle (°)	Moisture Content (%)	Water Vapor Permeability (×10^−9^ g·mm^−1^·h^−1^·Pa^−1^)
GG	84.24 ± 2.53 ^a^	26.11 ± 0.59 ^d^	3.09 ± 0.22 ^b^
GG/5%URH	85.12 ± 3.21 ^a^	20.30 ± 0.38 ^c^	2.91 ± 0.18 ^b^
GG/10%URH	85.53 ± 2.18 ^a^	21.56 ± 0.12 ^c^	3.00 ± 0.28 ^b^
GG/20%URH	95.72 ± 3.54 ^b^	18.02 ± 0.31 ^b^	2.51 ± 0.31 ^a^
GG/30%URH	99.46 ± 2.78 ^c^	15.20 ± 0.17 ^a^	2.41 ± 0.17 ^a^

Data are expressed as mean ± standard deviation (*n* = 3). Different superscripts (a, b, c, d) within the same column indicate significant differences (*p* < 0.05).

**Table 6:** Physical thickness and opacity of neat GG and GG/URH composite films.

Sample	Film Thickness (mm)	Film Opacity (mm^−1^)
GG	0.20 ± 0.06 ^a^	3.15 ± 0.10 ^a^
GG/5%URH	0.21 ± 0.07 ^a^	3.03 ± 0.12 ^a^
GG/10%URH	0.24 ± 0.04 ^b^	3.29 ± 0.21 ^a^
GG/20%URH	0.27 ± 0.04 ^c^	4.22 ± 0.28 ^b^
GG/30%URH	0.31 ± 0.03 ^d^	4.83 ± 0.14 ^c^

Data are expressed as mean ± standard deviation (*n* = 3). Different superscripts (a, b, c, d) within the same column indicate significant differences (*p* < 0.05).

## Data Availability

The original contributions presented in this study are included in the article. Further inquiries can be directed to the corresponding author.
